# Pluronic F127 and D-α-Tocopheryl Polyethylene Glycol Succinate (TPGS) Mixed Micelles for Targeting Drug Delivery across The Blood Brain Barrier

**DOI:** 10.1038/s41598-017-03123-y

**Published:** 2017-06-07

**Authors:** Xin Meng, Jiansheng Liu, Xiangrong Yu, Jiajia Li, Xiaotong Lu, Teng Shen

**Affiliations:** 1 Department of Pharmaceutics, Key Laboratory of Smart Drug Delivery, Ministry of Education, School of Pharmacy, Fudan University, 826 Zhangheng Road, Shanghai, 201203 China; 2 Department of Neurology, Shanghai Ninth People’s Hospital, Shanghai Jiao Tong University School of Medicine, 639 Zhizaoju Road, Shanghai, 200011 China; 3 Department of Radiology, Zhuhai People’s Hospital, Zhuhai Hospital of Jinan University, 79 Kangning Road, Zhuhai, 519000 China; 4 The Institutes of Integrative Medicine of Fudan University, 12 Wulumuqi Middle Road, Shanghai, 200040 China

## Abstract

A novel polymeric mixed micelle composed of Pluronic F127 and D-α-tocopheryl polyethylene glycol succinate (TPGS) was developed to improve the delivery of fluorescent dyes and protein across the blood brain barrier (BBB). Rhodamine 123 (Rho123) and DiR loaded mixed micelles, composed of Pluronic F127 and TPGS with proportion of 4:1 (FT), were prepared by thin-film hydration, and β-galactosidase (β-Gal) loaded FT mixed micelles were prepared by self-assembly. The brain-targeted capability of FT mixed micelles were evaluated both *in vitro* and *in vivo*. The FT mixed micelles showed that a average particle size of 20.03 nm, and a low CMC of 0.0031% in water. The *in vitro* release of Rho123 from Rho123 loaded FT mixed micelles (FT/Rho123) presented a sustained-release property. FT/Rho123 also showed higher efficiency for the accumulation in brain capillary endothelial cells (BCECs) and brain tissues. β-Gal, a model protein, was also delivered and accumulated efficiently in the brain by spontaneous loading in the FT mixed micelles. Therefore, the results indicated that F127/TPGS mixed micelles may be considered as an effective nanocarrier for the brain-targeted delivery of diagnostic and therapeutic drugs.

## Introduction

Blood brain barrier (BBB) is responsible for regulating the neural microenvironment^1^. The structure for the barrier mainly composed of brain capillary endothelial cells with tight junctions, which expresses efflux transport proteins that control the transport of substances into the brain and maintain homeostasis of central nerve system (CNS)^2, 3^. However, the BBB also constitutes a major restriction to penetration of therapeutic agents to CNS^4^. This specific property of BBB induce poor treatment effects of nearly 98% small-molecule drugs and almost all macromolecules for brain diseases such as brain tumors, epilepsy and Alzheimer’s disease^4, 5^. Therefore, discovery of new modalities allowing for effective drug delivery targeting to the brain is of great importance for treatment of CNS diseases^6^. Nanoparticle-based drug delivery systems including micelles, nanoparticles and liposomes, have shown great potential in overcoming BBB and transporting drugs directly to the brain^7^. Considerable efforts have been made to add targeting moieties or imaging contrast to the nanocarriers to enhance brain delivery over the past decade. However, additional functionality means additional synthetic steps and costs, more convoluted behavior and effects *in vivo*, and also greater regulatory hurdles^8^. Therefore, despite their enormous potential, their translation from bench to bedside has not been so successful as compared with non-targeted nanoparticles so far^9^. Besides, a recent study has found some commonly used moieties that targets on BBB actually did not facilitated significant antibody brain uptake^10^. Therefore, it is still of tremendous value to develop nano-based drug delivery systems which make the full use of the properties of nanomaterials while not fundamentally modifying the molecular structure of them (e.g. self-assembly)^9^.

Complex different nanomaterials of complementary properties seems to be one of the feasible ways to achieve that purpose. Zhang *et al*. showed that micelles composed of Pluronic P123 and F127, which combine the MDR sensitizing properties of P123 with the long circulation effect of P127, enhanced the activity of PTX to overcome MDR in lung cancer^11^. Liu *et al*. also showed that a tryptophan derivate functionalized Pluronic P123/F127 mixed micelles can be developed to promote AEDs delivery to brain by transporter-mediated endocytosis as well as overcoming MDR^12, 13^. Pluronic block copolymers consist of hydrophilic polyethylene oxide (PEO) and hydrophobic poly propylene oxide (PPO) segments arranged in a basic tri-block structure: PEO-PPO-PEO, which were widely used as micellar carriers^6, 14^. Besides their ability to self-assemble into micelles, Pluronic block copolymers such as Pluronic P85, L61, P123 have been shown to be able to inhibit multidrug transporters, e.g., P-glycoprotein (P-gp), for increasing the delivery of agents to the brain^15, 16^. Pluronic F127 (PEO_100_-PPO_69_-PEO_100_) which has relatively extended PEO blocks, is commonly used as an ideal micellar carrier for its low CMC (0.0031%, w/w), excellent biocompatibility and satisfactory safety^11^. It was used to enhance the encapsulation efficiency and improve the stability and circulation time of drugs^17^. However, F127 has little effect on P-gp efflux pump and practically do not transport across the membrane due to its relatively hydrophilic property. Thus, to make the full advantage of this material, as well as to overcome the shortcomings of Pluronic micelles described above, mixed micelles composed of F127 and another lipophilic copolymer with a biological modifying property warrant further investigation^15, 17^.

D-α-tocopheryl polyethylene glycol 1000 succinate (TPGS) is an onionic water soluble derivative of vitamin E, which is formed by conjugation of vitamin E succinate with polyethylene glycol (PEG)^18, 19^. It has also been approved by FDA as a safe pharmaceutical adjuvant used in diverse drug formulations^20^. Several advantages of TPGS make it available as a nanocarrier for drug delivery, including solubilization of poorly soluble drugs, enhancing the cellular uptake of the drugs and prolonging the blood circulation time of the drugs^18, 20^. Notably, TPGS has been served as a potent biological response modifier capable of inhibiting P-gp for decreasing efflux of drugs from cells and enhancing drugs transport across cellular barriers including brain endothelium^21^. However, the CMC of TPGS is relatively high (0.02%, w/w), which may make TPGS micelles dissociate in the plasma^20^. Therefore, it may be more appropriate to prepare mixed micelles with TPGS and other low CMC amphiphilic materials^18, 20^. Herein, we propose that Pluronic F127 and TPGS could be used for preparing mixed micellar carriers, which may combine the effects of two biomaterials and enhance the drug transport across the BBB.

In this study, fluorescent model probes and protein drug β-galactosidase (β-Gal) loaded F127/TPGS (FT) mixed micelles were prepared by simple methods. The mixed micelles were characterized in terms of critical micelle concentration, particle size distribution, morphological observation and *in vitro* release study. The *in vitro* and *in vivo* brain targeting capability of FT mixed micelles loading with fluorescent probe Rho123 were assessed using brain capillary endothelial cells (BCECs) and normal rats respectively. Both small-molecule fluorescent probe Rho123 and macromolecular model protein drug β-Gal, were used to evaluate the brain targeting ability of F127/TPGS mixed micelle-based nanocarriers.

## Results

### Characterization of F127 and TPGS mixed micelles

**Figure 1:**
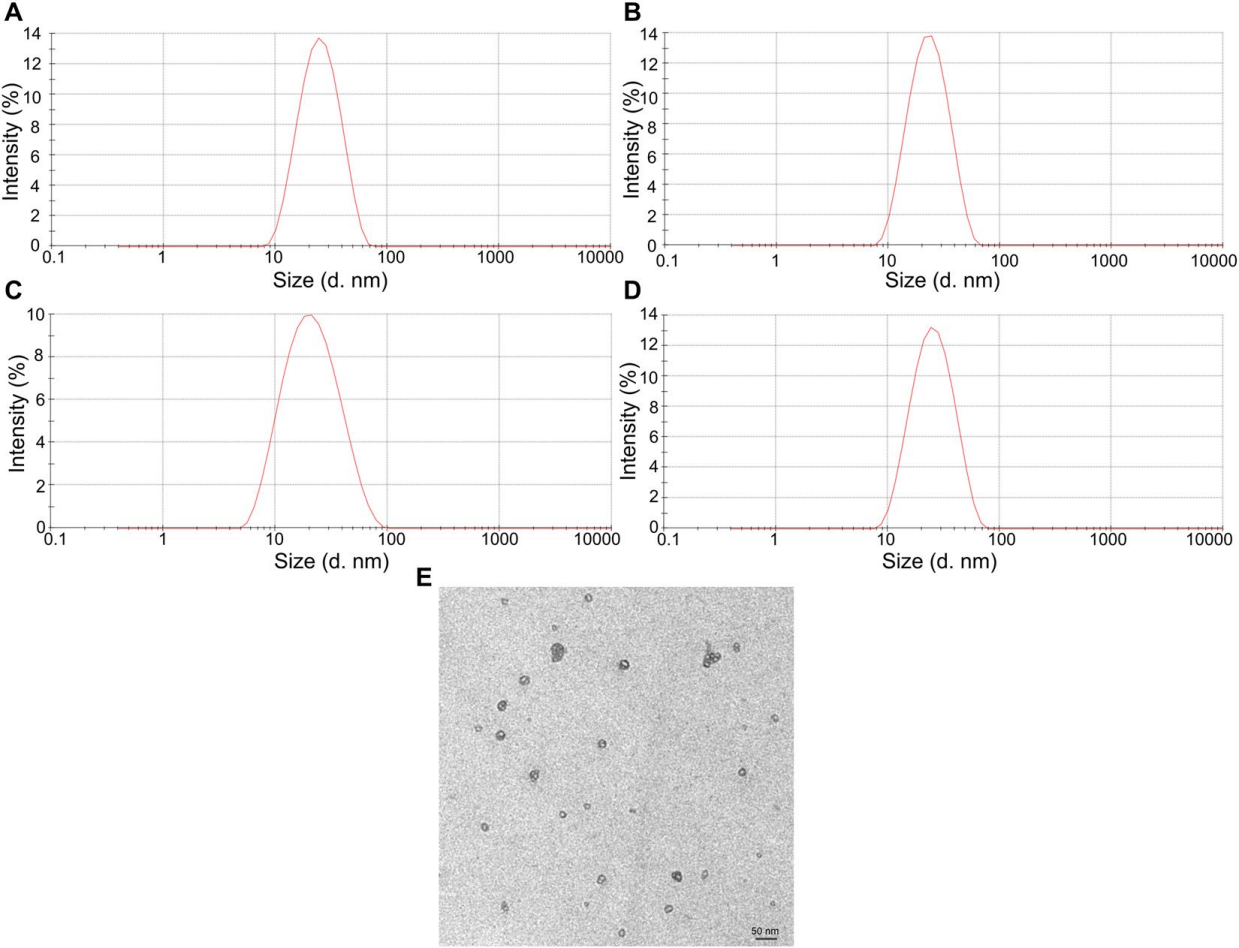
Particle size and size distribution of nanoparticles. Empty Pluronic F127 micelles (**A**); Empty FT micelles (**B**); DiR loaded FT micelles (**C**); Rho123 loaded FT micelles (**D**); TEM images of FT micelles (**E**). The scale bar represented 50 nm.

The average size and size distribution for empty micelles and drug loaded micelles were illustrated in Fig. 1. The average size of empty F127 micelles and mixed micelles composed of F127 and TPGS were 20.73 ± 0.16 nm (Fig. 1A) and 20.03 ± 0.55 nm (Fig. 1B), with poly dispersity index (PDI) 0.17 and 0.18 respectively. As seen from Fig. 1C,D, FT mixed micelles loading DiR or Rho123 did not visibly affect their size (19.19 ± 0.70 nm and 20.85 ± 0.98 nm, n = 3, *P* > 0.05) and size distribution (PDI = 0.16 and 0.2 respectively). The surface morphological study using TEM revealed that the mixed micelles were spherical in shape and moderate uniform in particle size (Fig. 1E). The results of the particle size measured by TEM and laser light scattering are in good agreement.

### CMC determination

**Figure 2:**
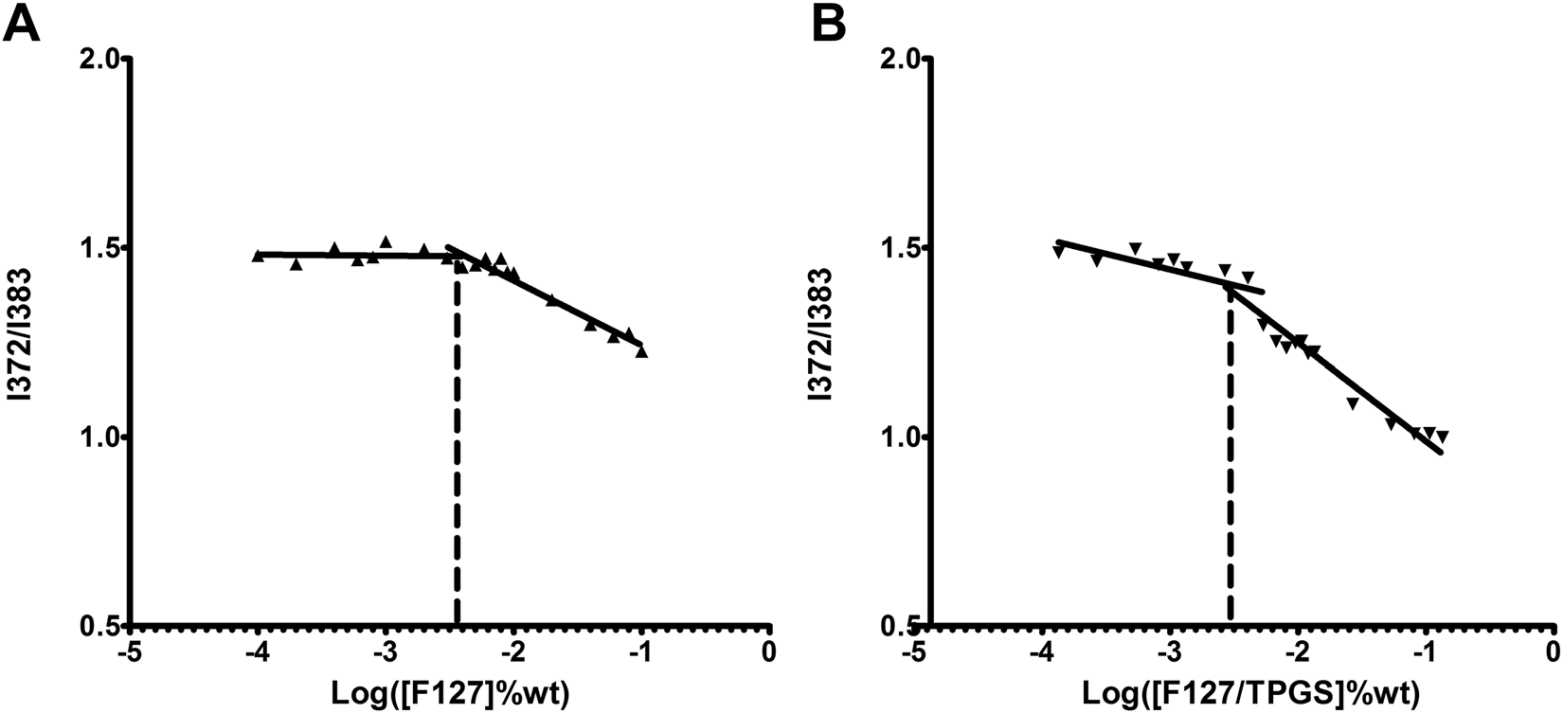
Plot of I372/I383 versus concentrations of copolymers in deionized water. Copolymers of F127 (**A**); F127/TPGS (4:1) (**B**).

In this study, the formation of micelles was measured by a fluorescence technique using pyrene as a hydrophobic probe. The emission spectra profile ratio of I372/I383 has been plotted versus polymer concentration shown in Fig. 2. The CMC value of F127 and FT mixture with the mass ratio of F127 to TPGS at 4:1 were shown to be ca. 0.0035% (Fig. 2A) and 0.0031% (Fig. 2B), which were compatible with previous reports^14^. The CMC of F/T mixture was even slightly lower than F127, indicating that addition of TPGS in micelles did not induced notable variation in the CMC of micelles.

### Rho123 *in vitro* release study

**Figure 3:**
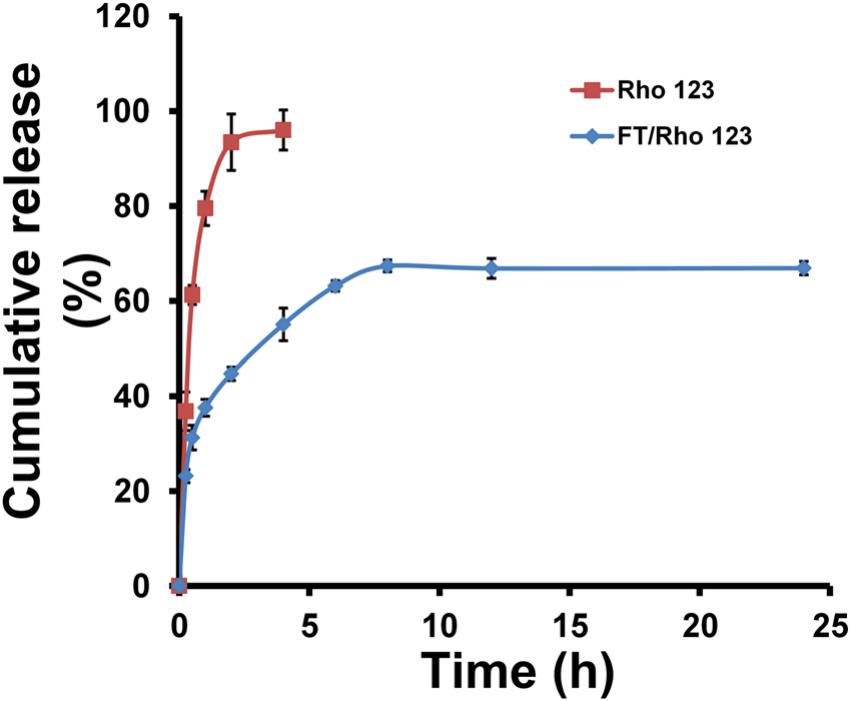
*In vitro* release of Rho 123-loaded FT mixed micelles. Free Rho123 dissolved in methanol was used as control. Each point represents mean ± SD (n = 3).

The *in vitro* release of Rho123 from control solutions and FT mixed micelles under sink condition was investigated by dialysis method with PBS (pH 7.4) as release medium. As shown in Fig. 3, almost all Rho123 was released from the methanol solution at the 4 h. During the same time period, only about 55% of Rho123 was released from FT/Rho123. After 24 h, 30–40% of the initially incorporated drug still existed in the micelles. The results indicated that the micelles showed sustained release as compared with free drug.

### Intracellular uptake of Rho123

**Figure 4:**
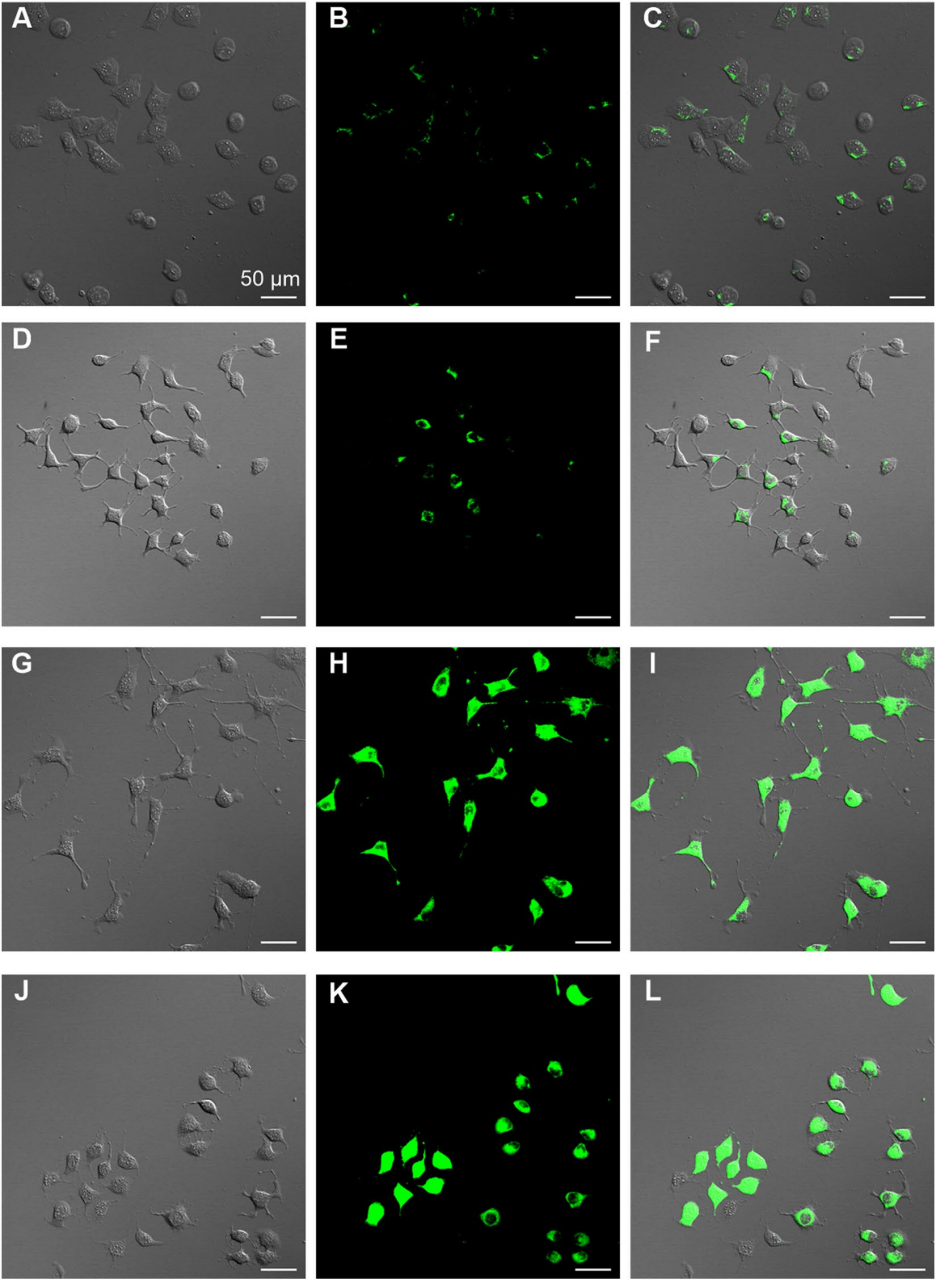
Effects of micellar carriers on the cellular uptake of Rho123 by BCECs. Bright field, fluorescence and merged images for BCECs incubated in free Rho123 (**A**–**C**), F127/ Rho123 (**D**–**F**), TPGS/Rho123 (**G**–**I**), FT/ Rho123 (**J**–**L**). Scale bar, 50 µm.

Rat brain endothelial cells (BCECs) were used in the cellular uptake study as an *in vitro* model of the BBB. Rho123, a traditional P-gp substrate, was used to evaluate the targeting effects of FT micelles across the BBB. Figure 4 shows confocal microscopy images of the BCECs obtained after 30 min incubation with the free Rho123 (Fig. 4A–C), Rho123-encapsulated micelles using different polymers with F127 (Fig. 4D–F), TPGS (Fig. 4G–I), F127/TPGS on mass ratio 4:1 (FT, Fig. 4J–L), respectively. To better compare the intensity of fluorescence between the cells treated with the free Rho123 and different Rho123-incorporated micelles, all images were recorded by the confocal microscope keeping the same laser intensity. It can be observed that the green fluorescence in the BCECs which corresponds to the Rho123-loaded TPGS (Fig. 4H) and FT (Fig. 4K) micelles were more conspicuous than that of the free Rho123 (Fig. 4B) while F127 (Fig. 4E) did not show remarkable increment of the fluorescence as compared with free Rho123.

### *In vitro* cell cytotoxicity

**Figure 5:**
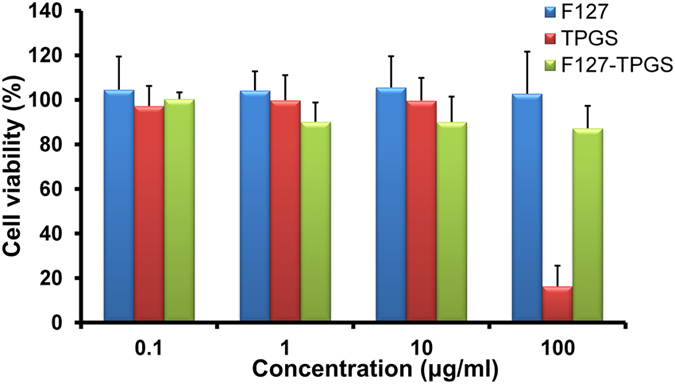
Viability of BCECs cells after 60 h treatment with varying concentrations of excipients (Pluronic F127, TPGS and FT mixed micelles). Data represent means ± SD (n = 6).

*In vitro* cytotoxicity of F127, TPGS and empty F127/TPGS (FT) mixed micelles was investigated with BCECs for 60 h incubation at 37 °C and the results were shown in Fig. 5. In the concentration ranges (0.1–100 μg/ml) used in this study, the cytotoxic effects of Pluronic F127 were negligible, as shown in Fig. 5 blue columns. This is consistent with previous research supporting that F127 can be suitably used as a micellar carrier with high biocompatibility. It was shown that TPGS displayed increasing cytotoxicity as the concentration increased from 10 μg/ml to 100 μg/ml (Fig. 5 pink columns), possibly due to the induced apoptotic properties of TPGS^22^. However, the cell viability for empty FT micelles at 0.1, 1, 10 and 100 µg/ml concentration was found to be 97.23 ± 9.00, 90.12 ± 8.68, 90.04 ± 11.41 and 87.19 ± 10.08%, respectively (Fig. 5 green columns), suggesting that the empty FT micelles achieved significantly lower cytotoxicity compared to TPGS at the identical concentration above 100 μg/ml. Therefore, the low toxic FT micelles used in this study showed a tremendous potential as a safe nanocarrier for drug delivery systems.

### *Ex vivo* optical imaging of brain and organs

**Figure 6:**
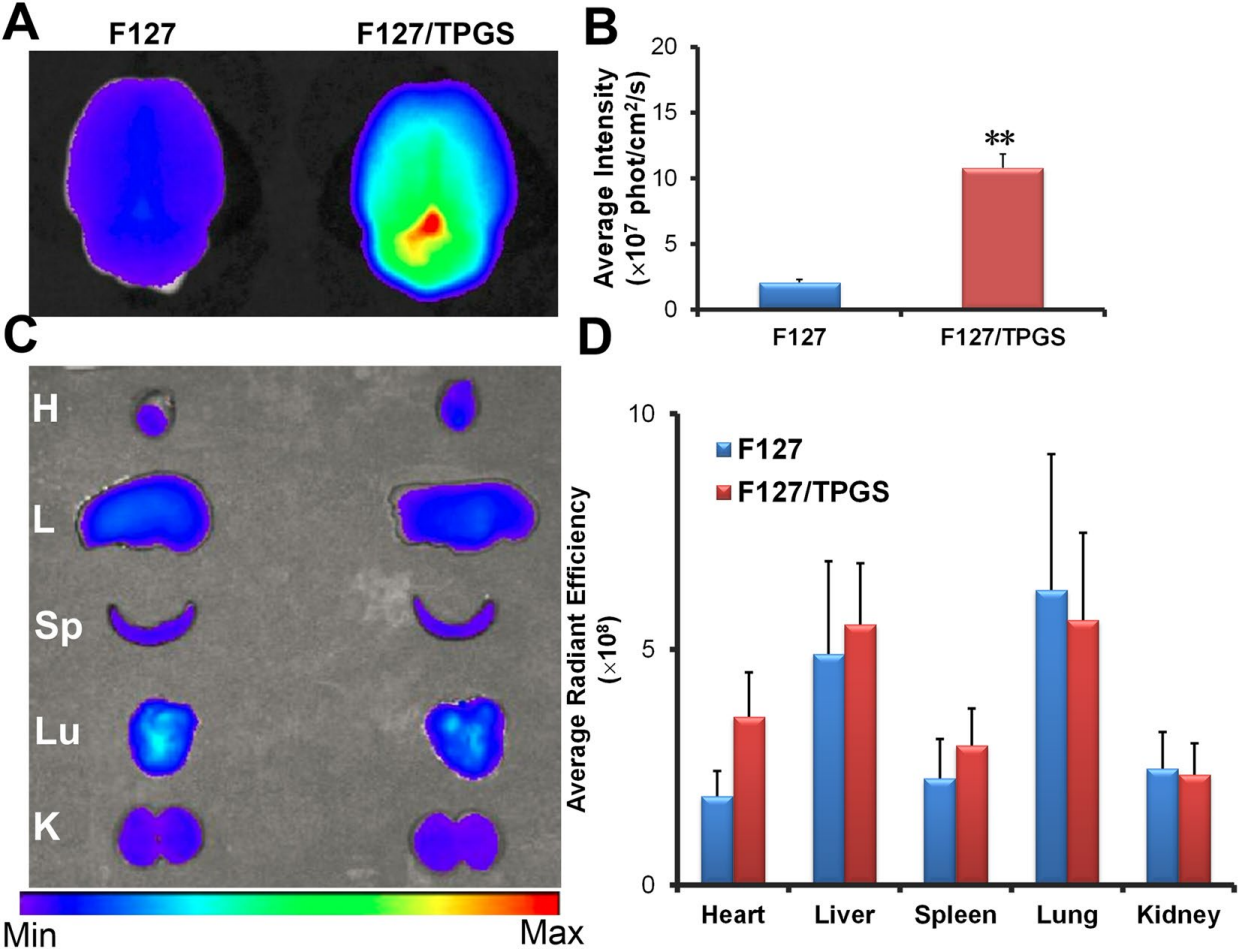
*Ex vivo* imaging of major organs post DiR-loaded micelles i.v. injection in rats. (**A**) *Ex vivo* imaging of the brains harvested at 2 h. (**B**) Semi-quantitative fluorescence intensity of brains. (**C**) *Ex vivo* imaging of other major organs (H: heart, L: liver, Sp: spleen, Lu: lung, K: kidney). (**D**) Semi-quantitative fluorescence intensities of those different organs. Statistically significant differences by *t*-test when compared to the corresponding value of the control. **P < 0.01, vs F127 (n = 3).

For brain targeted delivery, DiR loaded F127 and FT micelles were intravenously administered to rats. To obtain the distribution of DiR in major organs of rats, rats were sacrificed after 2 h injection and major organs including the heart, liver, spleen, lungs, kidney and brain were excised and the fluorescence of these organs were observed by IVIS imaging system (Fig. 6A and C). As shown in Fig. 6B, DiR loaded FT mixed micelles showed significantly higher intensity (~3.6 fold) from the brain than F127 micelles (*P* < 0.01, n = 3), demonstrating a high efficiency of FT mixed micelles for brain targeting. The body distribution of FT mixed micelles within other major organs were indistinctively altered as compared with F127 micelles (Fig. 6D). It has also been observed that the intensity signal associated with the liver and lung are stronger in comparison with that of the other tissues. These results indicated that FT micelles can contribute to BBB penetration and increase the transportation of fluorescent probe into the brain.

### Brain distribution of fluorescent-labeled micelles in rats

**Figure 7:**
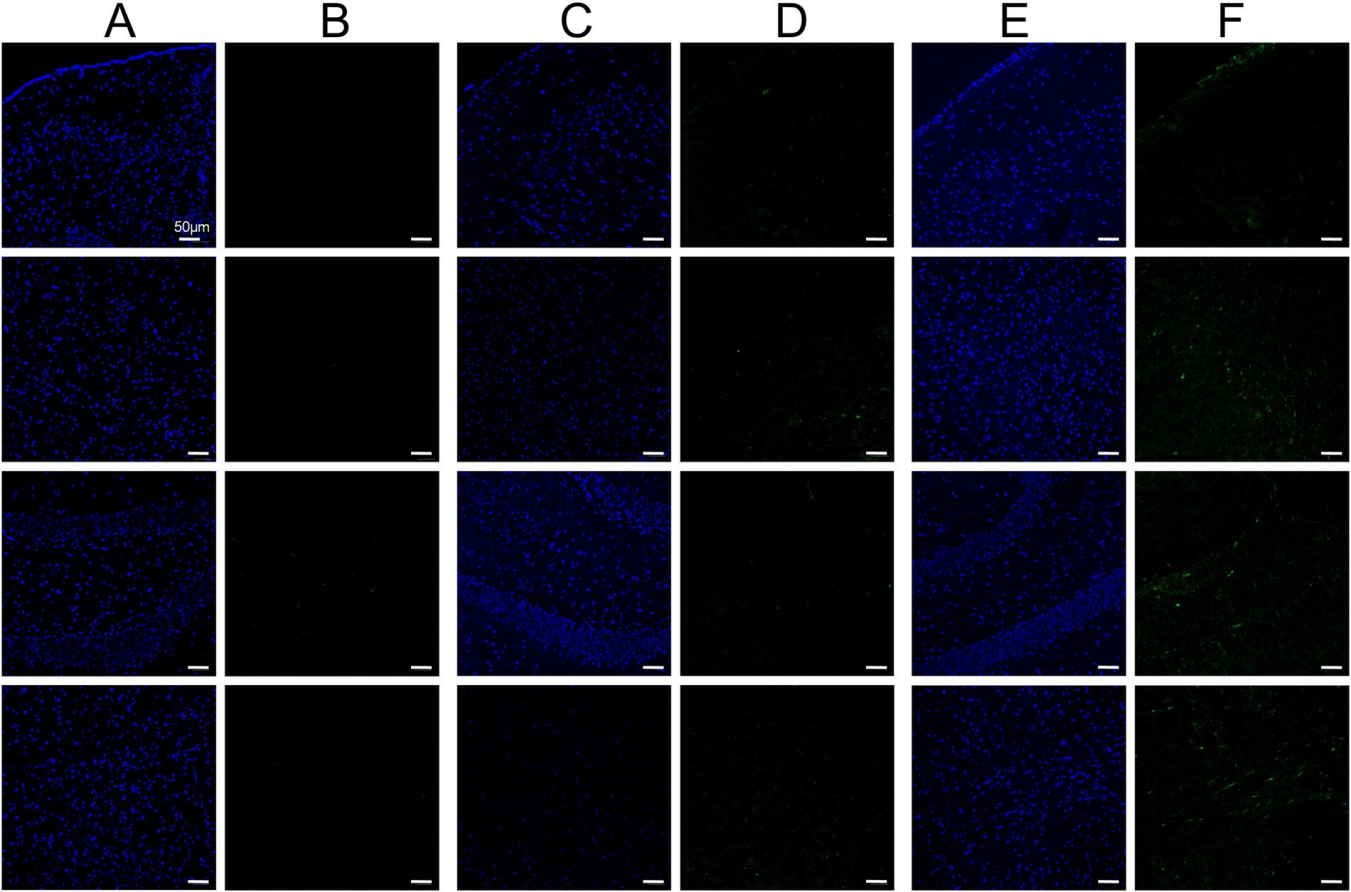
Confocal images of brain sections of rats treated with different formulations of Rho123. Free Rho 123 (columns **A**,**B**), Rho123- incorporated F127 (columns **C**,**D**) and FT mixed micelles (columns **E**,**F**) were i.v. injected separately. Rows 1–4 were images of cortex, caudate, hippocampus, substantianigra respectively. Blue fluorescence: cell nuclei, green fluorescence: Rho123. Scale bar, 50 µm.

Rho123 was also used to evaluate the brain targeting capability of micelles in rats. Figure 7 shows the confocal microscopy images of the sections of brain tissues including cortex, caudate, hippocampus and substantianigra after 2 h of intravenous injections free Rho123 or Rho123-incorporated micelles via tail vein. The nuclei were stained with Hoechst 33342 shown in blue fluorescence and the green fluorescence corresponds to the Rho123 present in the four regions of brains. As shown in Fig. 7 (columns B and F), accumulation of green fluorescence in the brain were much more abundant for FT mixed micelles than free Rho123. The green fluorescence associated with the brain sections clearly demonstrates the presence of FT mixed micelles in the brain, due to the FT mixed micelles were able to cross the BBB. Only slight increment of fluorescence was observed in F127 micelles when compared with free Rho123 (column D). However, the brighter signal in different regions of brains were observed for FT mixed micelles in comparison with F127 micelles (columns D and F). The analysis of the brain accumulation of nano-carriers including F127 and FT clearly suggests that FT were much more effective for the brain targeting. Therefore, FT mixed micelles was optimal for the targeted delivery to the brain herein.

### Histochemistry of β-galactosidase delivered to brain

**Figure 8:**
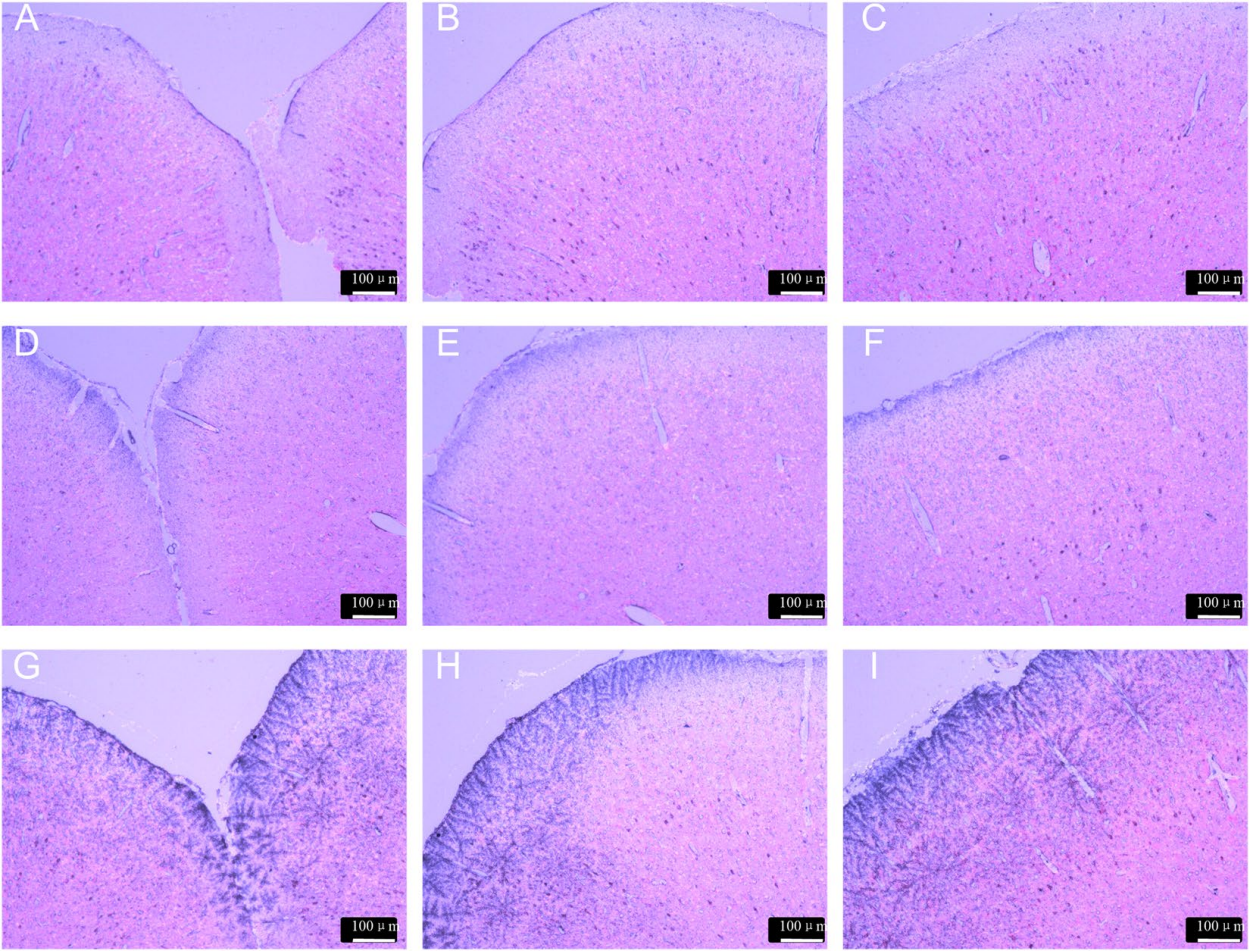
Analysis of β-gal enzyme activity assessed by β-Gal kit staining in the enlarged images. (**A**–**C**) β-gal saline; (**D**–**F**) β-gal-loaded F127 micelles; (**G**–**I**) β-gal-loaded FT mixed micelles (Scale bar: 100 μm).

The brain delivery of β-Gal as an enzymatic model drug incorporated in nanocarriers (F127 and FT mixed micelles) after intravenous administration in rats was assayed to evaluate the brain targeted efficacy of the carriers^23^. To determine whether the β-Gal delivered to the brain, the sections from rats brains were stained after 2 h i.v. injection of free β-Gal or nanocarriers as shown in Fig. 8. Intravenous administration of free β-Gal did not show any detectable enzymatic activity in the brain (Fig. 8A–C). When F127 micelles were injected after 2 h, the enzyme activities were indistinctively observed in comparison with free β-Gal (Fig. 8D–F). After injection of FT mixed micelles, the enzyme was clearly detected in the brain (Fig. 8G–I), and the enzyme activity was observed much stronger than free β-Gal as well as F127 micelles. The results clearly demonstrated that the FT mixed micelles could be effective as a brain targeting delivery vehicle for enzyme drug while maintaining its biological activity.

## Discussion

The treatment of CNS diseases such as brain cancers, epilepsy, CNS infection, Alzheimer’s and Parkinson’s diseases are confronted with big challenge due to the presence of BBB, which is a formidable obstacle for the delivery of most drugs to the brain^2, 23^. With the development of nanoparticles as brain-targeting delivery systems, certain drugs have been able to be transported directly into CNS, demonstrating the feasibility of the drug delivery to the brain using nano-based carriers^23, 24^. In this study, we report that Pluronic F127 and TPGS mixed micelles (FT) incorporating both small-molecule (Rho123) and macromolecule (β-Gal) model drugs can be readily prepared, which could efficiently enhance the brain penetration of these model drugs.

The major factor which influenced the drug delivery of copolymer micelles were the CMC, solubilization capacity and stability of carriers for the micelles^11^. Based on these reasons, Pluronic F127 and TPGS were used as micellar carriers with a higher proportion of F127. In preliminary experiment, we found the mixed micelles with the mass ratio of F127 to TPGS at 4:1 showed better stability compared with other ratios of 1:1, 3:2 and 9:1 (Fig. S1). Therefore, Pluronic F127 and TPGS on mass ratio of 4:1 was used in this study. The F127and TPGS (FT, 4:1 w/w) mixed micelles showed as uniform spherical particles under TEM. The micelle-core forming part probably attribute to hydrophobic PPO from F127 and vitamin E from TPGS respectively, while outer micelle-shell should respond to hydrophilic PEO in F127 and PEG in TPGS^25^. The size of empty or fluorescent dyes-incorporated micelles was about 20 nm, and it was showed that drug incorporation did not affect the micelles size distribution. Particle size plays a crucial role in determining bio-distribution and circulation times of the carriers *in vivo* ^18^. Particle size is considered to be one of the major parameters for efficient brain delivery of nano carriers^11, 26^. Small particle sizes (<200 nm) can avoid the recognition and reduce the uptake by the reticulo-endothelial system (RES) and prolong the half-life in the blood system resulting in passive targeting properties. In addition, small particles were suitable for intravenous administration^4^. Therefore, micelles prepared within asmall size may have the potential to be used effectively for enhanced permeability of drugs across the BBB.

The critical micelle concentration (CMC) is a essential parameter influencing the micelle’s stability *in vitro* and *in vivo* ^27^. One of the significant differences between the *in vitro* and *in vivo* conditions is the dilution effect under intravenous administration^10^. The CMC of pure F127 micelles is 0.0035% in our present study, in accordance with previous reports^11^. The CMC of F127/TPGS binary mixture is approximately 0.0031%. The low CMC value ensures the FT mixed micelles a high stability in the blood stream upon extreme dilution. The results showed the CMC of FT mixture was similar to that of pure F127, demonstrating that the dilution stability of FT mixed micelles was at least comparable to F127 micelles. The *in vitro* release results showed that 95% Rho123 in methanol solution were released within the first 2 h. This suggested that Rho123 can freely diffuse through the dialysis membrane, while 44.7% of Rho123 were released from the FT mixed micelles, which were much slower than free Rho123 solution. This result indicated that the micelle carrier can sustain the release of Rho123. The sustained-release mechanism might be related to drugs loaded as a separate phase inside the micellar core, subsequently inducing a slower drug release^16^.

Rho123, a small-molecular model drug as well as a fluorescent tracer dye of sub-cellular probes, has been used for the examination of membrane transport processes such as BBB^24, 28^. BCECs were used as an *in vitro* model for the cellular uptake study, which can form a tight monolayer and play an important role in maintaining the barrier properties of the BBB^29^. In present work, BCECs were incubated with the free Rho123 and the Rho123-load micelles with different carriers including F127, TPGS and F127/TPGS mixture. The poor uptake of free Rho123 were found in BCECs, which was consistent with previous report^16^; it was observed that TPGS could extremely increase the penetration of Rho123 in comparison with F127. Therefore, the difference on cellular uptake between these two carriers may be due to the variation in the physicochemical and biological properties^30, 31^. And the FT mixed micelles also had enhanced effects on penetration of Rho123 in BCECs which were similar to TPGS alone. This result indicated that the facilitative ability of FT mixed micelles on cellular uptake was mainly attributed to TPGS rather than F127. It has been reported in previous studies that the TPGS micelles could effectively enhance the cellular uptake possibly via a nonspecific absorption by the adsorptive-mediated endocytic pathway^20, 32^. Another reason for uptake enhancement of TPGS may be due to its biological modulatory property. TPGS has been proved as a reversal surfactant of P-gp, which predominantly exists on epithelia apical membranes involving BCECs, modulating efflux transport of drugs via P-gp ATPase inhibition^33–35^. In our research, we demonstrated that TPGS was an ideal biomaterial for mixed micellar formulation providing a high cellular uptake, so that FT mixed micelles could significantly enhance the uptake of Rho123 in BCECs.

To evaluate brain targeting capability of nano-carriers, DiR and Rho123 as model drugs loaded various nano-carriers were used by intravenous administration in rats. It was showed that DiR-incorporated FT mixed micelles achieved a higher brain accumulation in comparison with F127 micelles, while other organs such as heart, liver, spleen, lung and kidney did not show significant difference between the two groups. Further quantitative analysis showed that the average intensity of DiR for FT mixed micelles in brain tissue was more than 3-fold when compared with that of F127 micelles, demonstrating that FT mixed micelles have remarkable ability to improve the efficiency of DiR penetrating into the brain. Accumulation in other organs of FT mixed micelles and F127 micelles were almost comparable, which suggested that TPGS had negligible effect on pharmacokinetic *in vivo*. For brain distribution of Rho 123 in rats of various carriers, similar trends with DiR was observed. The significantly brighter fluorescence signal was observed for FT mixed micelles in comparison with free Rho 123 while slight signal of fluorescence was found in F127 micelles. Therefore, it was clearly demonstrated that both of the two model drugs incorporated FT mixed micelles was much more effective for brain accumulation than pure F127 micelles. It was interesting to note that the different accumulation of FT mixed micelles was observed in four regions (cortex, caudate, hippocampus, substantia nigra) of the rat brain. Fluorescence signal in cortex was much lower than that of the other three regions, which indicated that FT mixed micelles for brain delivery may decrease the CNS side effect of drugs when other three regions were main treatment targets^36^. The results also suggested an immense possibility that the FT mixed micelles transport in brain may be greatly increased in caudate, hippocampus and substantia nigra, which showed as a potential nanocarrier for treatment of CNS diseases, such as epilepsy or Alzheimer’s disease^13, 23^.

In previous studies, brain delivery of protein was investigated by using various carriers, such as PLGA particles and specific RVG modified pluronic-based nanocarrier^22, 37^. However, to facilitate the BBB transfer, these protein delivery methods were high-cost in complex synthesis and modification. The FT mixed micelles in this study were prepared by simple self-assembly method, resulting in an significantly increased brain targeting effect^2, 19^. β-Gal was used as a model enzyme drug to evaluate the efficacy of FT mixed micelles for delivery of protein to the brain. The brain delivery of β-Gal was observed by X-Gal staining of the brain sections. When i.v. injection with β-Gal incorporated FT mixed micelles, brains in rats showed tremendously stronger β-Gal activity compared with free β-Gal and F127 micelles. The results demonstrated that FT mixed micelles was also effective as a nanocarrier for enzyme drug which could delivery enzyme to the brain while maintaining its biological activity. Therefore, the FT mixed nanocarrier could be aslo used as a targeted vehicle for the brain delivery of proteins (e.g., enzymes or antibodies), thus for the treatment of brain disease.

## Conclusion

The mixed polymeric micelles, composed of Pluronic F127 and TPGS with the proportion of 4:1, exhibited a low CMC and a sustained-release property. The TEM analysis showed the nano range of the FT mixed micelles. Compared with free Rho123, Rho123-loaded FT mixed micelles showed higher cellular uptake efficiency on BCECs and more desired accumulation in the brains of rats after intravenous administration. FT mixed micelles was also effective for the delivery of β-Gal across the BBB. Therefore, the FT mixed micelle maybe a potentially promising nanocarrier for the brain targeted delivery vehicle of protein drugs as well as small-molecule drugs in the future.

## Methods

### Materials

Pluronic^®^ F127 (F127) was supplied by BASF Corporation (Ludwigshafen, Germany) without further purification. D-α-tocopheryl polyethylene glycol 1000 succinate (Vitamin E TPGS or TPGS) was purchased from Eastman Chemical Company (Kingsport, TN, USA). Rhodamine123 (Rho123) were purchased from Sigma-Aldrich Co. (StLouis, MO, USA). 1,10-Dioctadecyl-3,3,30,30- tetramethylindo-tricarbocyanine iodide (DiR), a near-infrared dye, was obtained from Biotium (Invitrogen, USA). β-galactosidase (β-Gal) was purchased from Meilune company (Dalian, China). Dulbecco’s Modified Eagle’s Medium (high glucose) (DMEM), fetal bovine serum (FBS), trypsine-EDTA (0.25%) and penicillin - streptomycin were purchased from Gibco (CA, USA). Cells counting kit-8 (CCK-8) were purchase from DOJINDO laboratories (Shanghai, China).

High performance liquid chromatography (HPLC) grade methanol were purchased from Merck KgaA (Darmstadt, Germany). All other solvents were analytical grade.

### Cells and animals

Rat brain capillary endothelial cells (BCECs) were obtained from the Chinese Academy of Sciences Cell Bank (Shanghai, China). Primary BCECs were cultured as described previously^13, 16^. Cells were expanded and maintained in special DMEM supplemented with 10% FBS, 100 IU/ml penicillin and 100 mg/ml streptomycin and cultured at 37 °C under a humidified atmosphere containing 5% CO_2_.

Sprague-Dawley (SD) rats (male, 200–250 g) were purchased from Sino-British SIPPR/BK Lab Animal Ltd. (Shanghai, China). Rats were maintained at the control temperature of 23 ± 1 °C, humidity of 60 ± 5%, in a 14 h light/10 h dark cycle and provided with foods and water freely. All animal experiments were performed in accordance with guidelines and regulations evaluated and approved by the ethics committee of Fudan University.

### Preparation of F127 and TPGS mixed micelles

F127 and TPGS empty micelles or fluorescent dyes loaded micelles were prepared by thin-film hydration method^38^. Briefly, 80 mg micellar carriers composed of F127 and TPGS with different proportions and different fluorescent dyes were dissolved in 5 ml methanol. Then the organic solvent mixture was transferred to anround-bottom flask and evaporated under high vacuum to produce a thin film of polymers. The film obtained was further dried under vacuum overnight to remove any residual solvents. After that, the film was hydrated in 2 ml deionized water under 50 °C with stirring at 200 rpm for 1 h to form a micellar suspension. The suspension was filtered through a 0.22 μm filter membrane to obtain a clear solution of micelle.

To obtain proteins loaded micelles, proteins were efficiently loaded into nanocarriers by co-incubation at low temperature. F127 or F127/TPGS was mixed with 1 ml aqueous solution containing 50 μg β-Gal, and then incubated at 4 °C for 12 h to induce spontaneous loading of the protein into the nanocarrier.

### Characterization of F127 and TPGS mixed micelles

The size and distribution of micelles were measured using a dynamic light scattering spectrometer (Malvern Zetasizer Nano ZS; Malvern Instruments, Worcestershire, UK). The samples were analyzed without dilution. Measurements were repeated three times for each solution and the record was an average of three times values.

The morphology of the micelles was observed under transmission electron microscope (TEM; Philips CM 120, Amsterdam, the Netherlands). A drop of micellar solution was placed on a copper grid covered a carbon film and stained by phosphotungstic acid solution (2%, w/v). After dying at room temperature, the sample was observed under the TEM.

### Critical Micelle Concentration (CMC)

The CMC values were obtained by a fluorescence probe technique using pyrene as a hydrophobic probe, as described previously^39^. Pyrene was added to a flask and dissolved in acetonitrile, then the solvent was evaporated. After that, a series of micellar solutions were added to tubes and the final pyrene concentration was maintained 6.0 × 10^−7^ M. The solution was incubated overnight in the dark and the concentration of encapsulated pyrene in micelle was measured using cary eclipse fluorescence spectrophotometer (Cary Elipse, Agilent Technologies, Santa Clara, CA, USA). Excitation wavelength was 334 nm and the fluorescence emission spectra was scanned between 350 nm and 500 nm. Fluorescence absorption intensity at 372 (I372) and 383 nm (I383) were recorded. CMC was obtained by plotting the ratio of I372/I383 of the emission spectra profile versus the logarithm of the copolymers mass concentration.

### Micelle stability

In order to assess the physical stability of the FT mixed micelles, DiR-loaded F127/TPGS mixed micelles with the ratio of 1:1, 3:2, 4:1 (FT) and 9:1 were incubated in a temperature controlled oven at 37 °C for 48 h. At predetermined time intervals, each sample was filtered through a 0.22 μm filter membrane followed by dilution with methanol. The concentration of DiR remaining in solution was measured using fluorescence spectrophotometer (Cary Elipse, Angilent, USA).

### Rho123 *in vitro* release study

The *in vitro* release behaviors of Rho123 from FT mixed micelles were investigated by dialysis method with PBS (pH 7.4) as release medium^13^. The micellar solution containing Rho123 (4 μg/ml, 0.5 ml) was introduced into a pre-swollen dialysis bag (Moleculeweight cut-off, MWCO = 2.0 kDa), and the end-sealed dialysis bag was immersed into 40 ml release medium at 37 °C with stirring speed at 100 rpm. At predetermined time intervals (0, 0.25, 0.5, 1,2, 4, 6, 8, 12, 24 h), 3 ml of the dissolution medium was withdrawn and the same volume of fresh medium was added. The amounts of Rho 123 were detected by fluorescence spectrophotometer (Cary Elipse, Angilent, USA). For comparison, the release of Rho 123 from methanol solution was conducted under the same conditions.

### Intracellular uptake of Rho123

For the cellular uptake study, BCECs were seeded at the density of 10^4^ cells in cell culture dishes (35 mm × 20 mm; Corning-Coaster, Tokyo, Japan). After incubated for 48 h, the cells were observed under the microscope for morphology. BCECs were incubated in free Rho 123 solution (1 mM in DMSO and diluted to 5 μM with PBS) or equivalent Rho123 mixed with different polymeric micellar solutions (F127, TPGS or F127/TPGS mixed solution on mass ratio 4:1, 0.05% w/v) at 37 °C for 30 min. Then the cell dishes were washed with cold PBS for four times briefly. The fluorescence of cells was monitored by using confocal laser scanning microscope (Carl Zeiss, LSM 710, Oberkochen, Germany) immediately.

### *In vitro* cell cytotoxicity

For cytotoxicity measurements, cells were seeded in 96-well plates at the density of 10^4^ cells per well (100 μl volume) and incubated at 37 °C with 5% CO_2_ for 24 h. After that, the growth medium was replaced with 100 μl fresh medium solution containing different excipients (Pluronic F127, TPGS and blank FT mixed micelles) with concentration ranging from 0.1 to 100 μg/ml for 60 h. Cell viability was measured using cell counting kit-8 (CCK-8) assay. 10 μl of CCK-8 solution were added to each well and the plate was incubated for 2 h. The absorbance of each well was measured by the microplate reader (BioTek Synergy 2, BioTek Instruments, VT, USA) with a wavelength of 450 nm.

### *Ex vivo* optical imaging of brain and organs of rats

The optical images were visualized by using the Caliper IVIS Spectrum Imaging System (PerkinElmer, MA, USA). Rats were intravenously injected with DiR-incorporated F127 micelles and F127/TPGS (4:1, w/w) mixed micelles (40 μg DiR/kg) through the tail vein. To get the distribution of micelles in brain and major organs, rats were sacrificed at 2 h post injection of micelles. And the fluorescence images of organs were obtained.

### Brain distribution of fluorescent-labeled micelles in rats

Free Rho123 and Rho 123-incorporated F127 micelles and F127/TPGS (4:1, w/w) mixed micelles were administered into rats via tail vein (Rho123 3.5 mg/kg of body weight). Two hours after injection of Rho123, animals were anaesthetized with 10% (w/v) chloral hydrate and hearts were perfused with 150 ml saline, followed by 100 ml 4% paraformaldehyde fixative. The whole brain was removed and fixed in 4% paraformaldehyde for 48 h and immersed in 15% sucrose PBS solution for 24 h until subsidence, then the brain samples were placed into 30% sucrose solution for 48 h. After that, excised brains were frozen at −80 °C in optimum cutting temperature (OCT) embedding medium (Sakura, Torrance, CA, USA). Coronal brain sections of cortex, caudate, hippocampus and substantianigra were obtained according to Paxinos and Watson^36^. Frozen sections of 10 μm thickness were prepared with acryotome Cryost at (Leica, CM1900, Wetzlar, Germany). The sections were placed at room temperature and rinsed with phosphate buffer solution (PBS, pH 7.4) two times before staining with1 μg/ml Hoechst 33342 solution for 5 min. Then after washing with PBS four times for 5 min, the slices were imaged under the confocal fluorescence microscope.

### Histochemistry of β-galactosidase delivered to brain

Rats were injected with β-Gal incorporated micelles (F127 and FT) and the control group was received injection of β-Gal saline (β-Gal 130 μg/kg of body weight). After two hours, rats were sacrificed and brains were removed. 10-μm thick frozen coronal brain sections were prepared in the way described above. β-Gal delivered to brain was characterized by *in situ* β-Gal Staining Kit (Bytotime company, Shanghai, China). The brain sections were fixed in fixative solution for 10 min, then washed with PBS three times for 5 min each time. After that, the sections were incubated in X-gal staining solution at 37 °C for 24 h covering by sealing membrane to prevent from drying. The brain sections were washed with PBS three times for 5 min. To counter stain, sections were incubated with neutral red solution for 15 seconds, and then washed with PBS solution briefly. Then the sections were monitored by using a fluorescence microscope (DMI4000B, Leica, Germany).

### Statistical analysis

The data are expressed as plus or minus the standard error of the mean (±SD). The statistical differences between the values of two groups were analyzed using the non-paired *t*-test. Avalue of *P* < 0.05 was considered to be statistically significant. The Statistica software (SPSS statistics 17.0) was used for performing statistical analyses.

## Electronic supplementary material

Supplementary information
