# Supplementary material for: Pluronic F127 and D-α-Tocopheryl Polyethylene Glycol Succinate (TPGS) Mixed Micelles for Targeting Drug Delivery across The Blood Brain Barrier
Source: Sci Rep. 2017 Jun 7;7:2964. doi: 10.1038/s41598-017-03123-y (PMC5462762; doi:10.1038/s41598-017-03123-y)
Supplement: Supplementary file 1 — Supplementary information [file 41598_2017_3123_MOESM1_ESM.pdf]

Pluronic F127 and D-α-Tocopheryl Polyethylene Glycol Succinate (TPGS) Mixed Micelles for Targeting Drug Delivery across The Blood Brain Barrier

Xin Meng[1*], Jiansheng Liu[2*], Xiangrong Yu[3*], Jiajia Li[1], Xiaotong Lu[1], Teng Shen[1,4]

[1]Department of Pharmaceutics, Key Laboratory of Smart Drug Delivery, Ministry of Education, School of Pharmacy, Fudan University, 826 Zhangheng Road, Shanghai 201203, China

[2]Department of Neurology, Shanghai Ninth People's Hospital, Shanghai Jiao Tong University School of Medicine, 639 Zhizaoju Road, Shanghai 200011, China

[3]Department of Radiology, Zhuhai People's Hospital, Zhuhai Hospital of Jinan University, 79 Kangning Road, Zhuhai 519000, China

[4]The Institutes of Integrative Medicine of Fudan University, 12 Wulumuqi Middle Road, Shanghai 200040, China

[*]These authors contributed equally to this work.

Corresponding author: Teng Shen, Department of Pharmaceutics, Key Laboratory of Smart Drug Delivery, Ministry of Education, School of Pharmacy, Fudan University; The Institutes of Integrative Medicine of Fudan University.

Address: 826 Zhangheng Road, Shanghai 201203, China; 12 Wulumuqi Middle Road, Shanghai 200040, China.

Tel.: +86-021-51980081; fax: +86-021-51980081.

E-mall: shenteng@fudan.edu.cn.

Supplementary data.

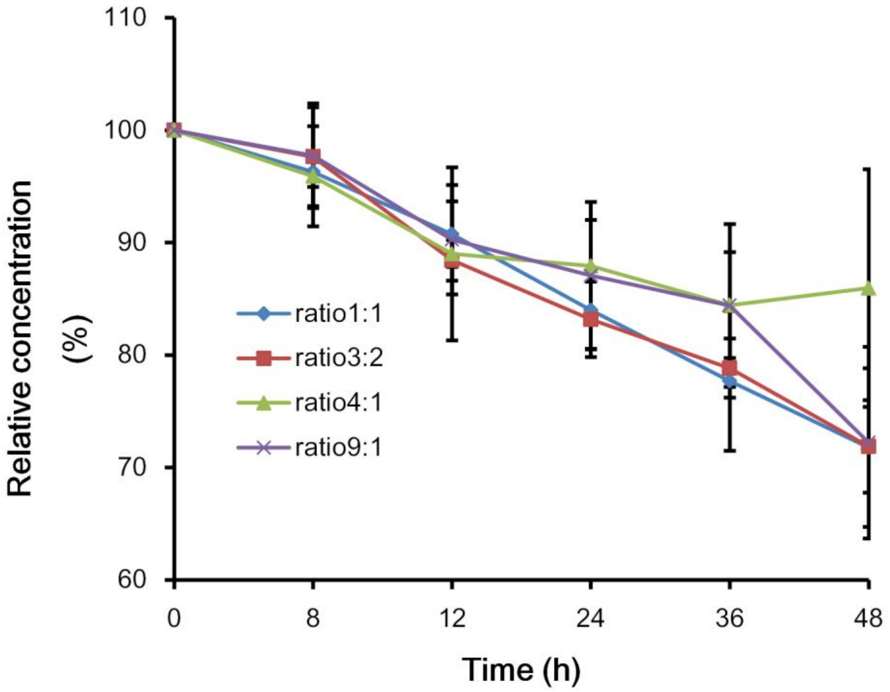

Figure S1. The stability of DiR-loaded F127/TPGS mixed micelles with different ratios incubated at 37 ℃. Each point represents mean ± SD (n = 3).
